# Local anesthetic overdose during nurse-led epidural analgesia due to occlusion alarm misinterpretation: a case report and literature review

**DOI:** 10.3389/fmed.2026.1854450

**Published:** 2026-08-12

**Authors:** You Yuan, Hong Zhou, Feiyu Yan, Wenbo Chen, Rujun Hu

**Affiliations:** 1 Department of Critical Care Medicine, Affiliated Hospital of Zunyi Medical University, Zunyi, Guizhou, China; 2 Department of Critical Care Medicine, Zhejiang Provincial People's Hospital Bijie Hospital, Bijie, Guizhou, China

**Keywords:** medication errors, anesthesia, epidural, adverse effects, patient safety, infusion pumps

## Abstract

**Rationale:**

Epidural analgesia (EA) medication errors are rare but serious adverse events in anesthesia and pain management. Previous literature has predominantly reported errors involving drug types or routes of administration, whereas those caused by infusion devices are uncommon. This case highlights the potential hazard of dose overdose resulting from an abnormal “occlusion” alarm when using a syringe pump for EA bolus.

**Patient concerns:**

A 36-year-old male with recurrent hyperlipidemic severe acute pancreatitis, third episode, was admitted to the intensive care unit (ICU). On admission, the pain score was 7/10 (visual analog scale, VAS). Thoracic epidural analgesia (T8-T9) was administered, with continuous infusion and intermittent boluses of a local anesthetic mixture for pain relief.

**Diagnosis:**

Extensive sympathetic blockade due to EA local anesthetic overdose, manifested as acute hypotension, tachycardia, and bilateral lower limb numbness after EA bolus administration.

**Interventions:**

Given that the patient's pain score was as high as 8/10 on day 4, a 5 ml EA bolus of the analgesic mixture was prescribed. The nurse used a syringe pump for the bolus. Due to high catheter resistance, the pump repeatedly triggered an “occlusion” alarm. The nurse misinterpreted this as no drug infusion and continued the procedure after repeatedly clearing the alarm, ultimately administering approximately 10 ml. Upon recognition, the EA route was immediately closed, and rapid intravenous infusion of 500 ml of compound sodium chloride solution was administered, along with intravenous norepinephrine at 0.2 μg/kg/min via a syringe pump.

**Outcomes:**

Hemodynamics recovered within 10 min, and sensation in both lower limbs gradually returned to normal within 30 min. The patient was transferred to another department 5 days after the event, with no long-term sequelae.

**Lessons:**

Misinterpretation of a syringe pump's “occlusion” alarm during nurse-led epidural bolus administration can lead to local anesthetic overdose. This case underscores the need for improved training on device characteristics and alarm management.

## 1 Introduction

The pain mechanism in severe acute pancreatitis (SAP) is complex. If not promptly controlled, pain can exacerbate pancreatic injury, leading to complications such as hemodynamic instability, hypoxia, and acute respiratory distress syndrome (ARDS), thereby delaying recovery (1, 2). Commonly used opioids may cause adverse effects including respiratory depression, drowsiness, constipation, and immunosuppression, while nonsteroidal anti-inflammatory drugs (NSAIDs) may induce gastrointestinal complications.

Thoracic epidural analgesia (TEA) involves the injection of low-concentration local anesthetics at the thoracic level (T8–T11) to block regional nerve roots, providing reversible control of sympathetic nerve activity. TEA not only offers effective analgesia and anti-inflammatory effects, but also promotes visceral vasodilation, improves organ perfusion, and reduces the incidence of complications such as intestinal paralysis (3, 4). Compared with intravenous sufentanil, TEA has a faster onset and provides more satisfactory pain relief within the first 24 h, and may reduce all-cause mortality in SAP patients (5).

Pain management in the ICU is regarded as the “fifth vital sign,” highlighting its growing importance. Nurses, as the healthcare providers most closely interacting with patients, play a leading role in pain assessment and management. Nurse-led epidural analgesia emphasizes proactive pain assessment, collaboration with physicians, and dynamic adjustment of analgesic strategies to achieve individualized pain control (6). However, insufficient familiarity with infusion devices and lack of standardized procedures may increase the risk of medication errors.

Medication errors are a major cause of iatrogenic error worldwide, with studies showing they can result in the death of 1 in 131 outpatients and 1 in 854 inpatients (7). Although the incidence of errors in neuraxial drug administration is relatively low, the consequences are often severe. Reviews have reported that more than 50 drugs have been inadvertently injected into the epidural space (8), while cases of overdose caused by using an intravenous infusion pump for epidural administration are rare, particularly in the context of nurse-led analgesia management. Patients and their families generally expect transparent communication and full disclosure when errors occur; even when no adverse outcomes result, most want to be informed of the error (9). This highlights the importance of improving medication safety, establishing standardized procedures, and ensuring that nurses are well-trained in device operation and risk recognition, especially under a nurse-led analgesia model.

This report describes a case in nurse-led EA management in which an intravenous infusion pump was used to administer analgesics through an epidural catheter. Repeated “occlusion” alarms were misinterpreted as failed drug delivery, leading to continued infusion. This resulted in local anesthetic overdose, hypotension, and bilateral lower limb numbness, highlighting the lack of a systematic training program for nurses and the reality that physicians, due to time constraints and other medical oversight responsibilities, have limited ability to provide such training.

## 2 Patient information

The patient was a 36-year-old man (168 cm, 88 kg; BMI 31.2 kg/m^2^) admitted with a 10-day history of abdominal pain and nausea. He was diagnosed with hypertriglyceridemia-induced SAP and admitted to the intensive care unit (ICU). On admission, he was fully conscious, with an APACHE II score of 10. His chief complaint was abdominal pain, with a pain score of 7 on the visual analog scale (VAS).

Vital signs were: blood pressure 135/82 mmHg, heart rate (HR) 105, respiratory rate (RR) 32, and temperature 37.8 °C. He had undergone open appendectomy 10 years earlier, and this was his third episode of SAP. He denied a history of hypertension, diabetes, cardiac disease, malignancy, or cerebrovascular disease, as well as infectious diseases such as hepatitis, typhoid, or tuberculosis. He had no drug allergies or family history of genetic disorders. The patient reported a smoking history of over 15 years and denied alcohol consumption.

**Figure 1:**
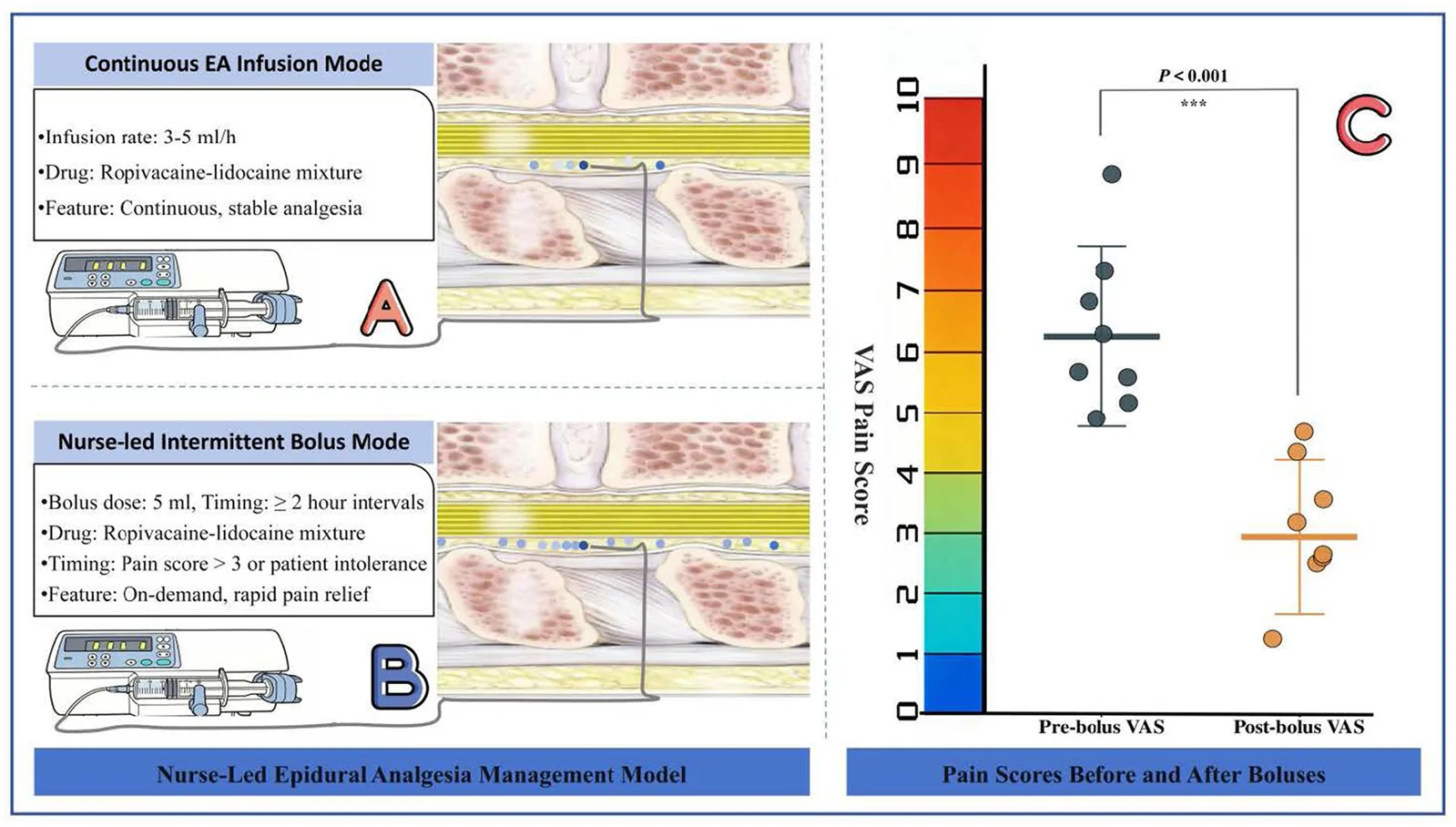
Nurse-led epidural analgesia model and pain scores. **(A)** Continuous infusion protocol; **(B)** Intermittent bolus protocol with ≥2-h intervals; **(C)** Pain scores before and after boluses.

On the day of admission, TEA was initiated, and an epidural catheter was placed for pancreatitis-related pain due to a high pain score of 7/10. Analgesia was managed using two approaches (Figure 1): a continuous infusion of 3–5 ml/h of a mixed solution (40 mg nalbuphine, 150 mg ropivacaine, 0.2 g lidocaine, diluted with 0.9% saline to 50 ml) provided stable baseline analgesia (Figure 1A), while a nurse-led intermittent bolus mode was applied as needed when the patient's pain score exceeded 3, delivering 5 mL boluses at ≥2-h intervals for rapid, on-demand relief (Figure 1B). This regimen was effective during initial catheter placement, although the patient's pain fluctuated, necessitating supplemental intermittent boluses.

## 3 Timeline

On the day of admission, an epidural catheter was placed at the T8-T9 interspace without complications. After successful placement, the anesthesiologist administered a test dose and initial analgesia of 5 mL of 2% lidocaine, equivalent to 0.1 grams, via the epidural catheter. The patient's pain score decreased from 7 to 2 on the visual analog scale. Continuous infusion of the mixed local anesthetic solution was then initiated at a rate of 3–5 ml/h. During catheterization, under nurse-led pain management, intermittent boluses of 5 ml of the same solution were administered via the infusion pump according to physician orders when the patient reported pain, with intervals of at least 2 h. Between the first and fourth day, 8 intermittent boluses were administered. Pain scores decreased significantly after each bolus, from 6.1 ± 1.5 before the bolus to 2.9 ± 1.2 after, with a paired *t* = 7.23, *P* < 0.001. Pain score changes before and after boluses are shown in Figure 1C. All boluses were administered smoothly without pump alarms or adverse events. The device used in this case was a conventional older-model intravenous syringe pump with a minimum infusion rate of 0.1 mL/h and both continuous infusion and bolus modes, and its occlusion alarm threshold was set to the standard value for intravenous infusion; this device did not have data logging capabilities.

On the afternoon of the 4th day, prior to the adverse event, the patient's vital signs were stable, with an invasive arterial blood pressure (ABP) of 129/80 mmHg, HR 110, RR 22, T of 36.8 °C, and oxygen saturation (SpO_2_) 97%. He was fully conscious, with normal motor and sensory function of both lower limbs and muscle strength graded 5. The epidural catheter was well secured, the puncture site showed no redness or exudation, and the infusion line was patent with continuous administration at 5 ml/h. The patient reported inadequate pain control, with a VAS score of 8. Physician orders were given for a single bolus of 5 ml of the analgesic solution via the epidural catheter. The attending nurse, a male with 2 years of ICU experience, used an intravenous infusion pump to administer the solution through the epidural catheter. The same 50 #mL syringe was used for both continuous infusion and bolus delivery. The pump repeatedly triggered occlusion alarms, which the nurse misinterpreted as failed drug delivery. The patient's pain did not improve, so he continued to override the alarms and push additional doses, during which he did not closely inspect the syringe markings. The estimated total bolus was approximately 10 mL, delivered over less than 5 min.

Approximately 5 min after administration, the patient developed progressive hemodynamic instability. ABP dropped to 80/40 mmHg, HR increased to 130, RR was 24, and SpO_2_ remained at 97%. The epidural infusion was immediately stopped. Other potential causes such as intracranial hemorrhage, hypoglycemia, and pulmonary embolism were excluded. The patient remained conscious but appeared lethargic and slightly drowsy, reporting dizziness. Neurological examination was performed. Sensory testing from the level of the nipples downward using a cotton swab revealed normal sensation above the nipple level and diminished sensation below, including reduced touch and pinprick perception. The sensory block corresponded with reduced muscle strength in both lower limbs, graded 3, and tendon reflexes were present but diminished. Upper limb sensory and motor function remained normal. No respiratory depression, altered consciousness, or seizures were observed.

According to physician orders, rapid intravenous infusion of 500 mL of compound sodium chloride solution was administered to expand blood volume. Norepinephrine was continuously infused via pump at 0.2 μg/kg/min to counteract vasodilation caused by sympathetic blockade and to correct hypotension. Vital signs and neurological function were closely monitored throughout the intervention.

## 4 Follow-up and outcomes

Approximately 10 min after the overdose event on the afternoon of day 4, the patient's hemodynamics recovered with ABP rising to 110/52 mmHg and HR stabilizing at 118. Norepinephrine infusion was gradually tapered over the next 20 min until discontinuation. Thirty min after the event, sensory function in both lower limbs gradually returned to normal. The patient tolerated fluid replacement and vasoactive therapy well, with no adverse reactions. Subjective symptoms, including dizziness and lower limb numbness, resolved, and the patient expressed satisfaction with the timely intervention by the medical team.

**Figure 2:**
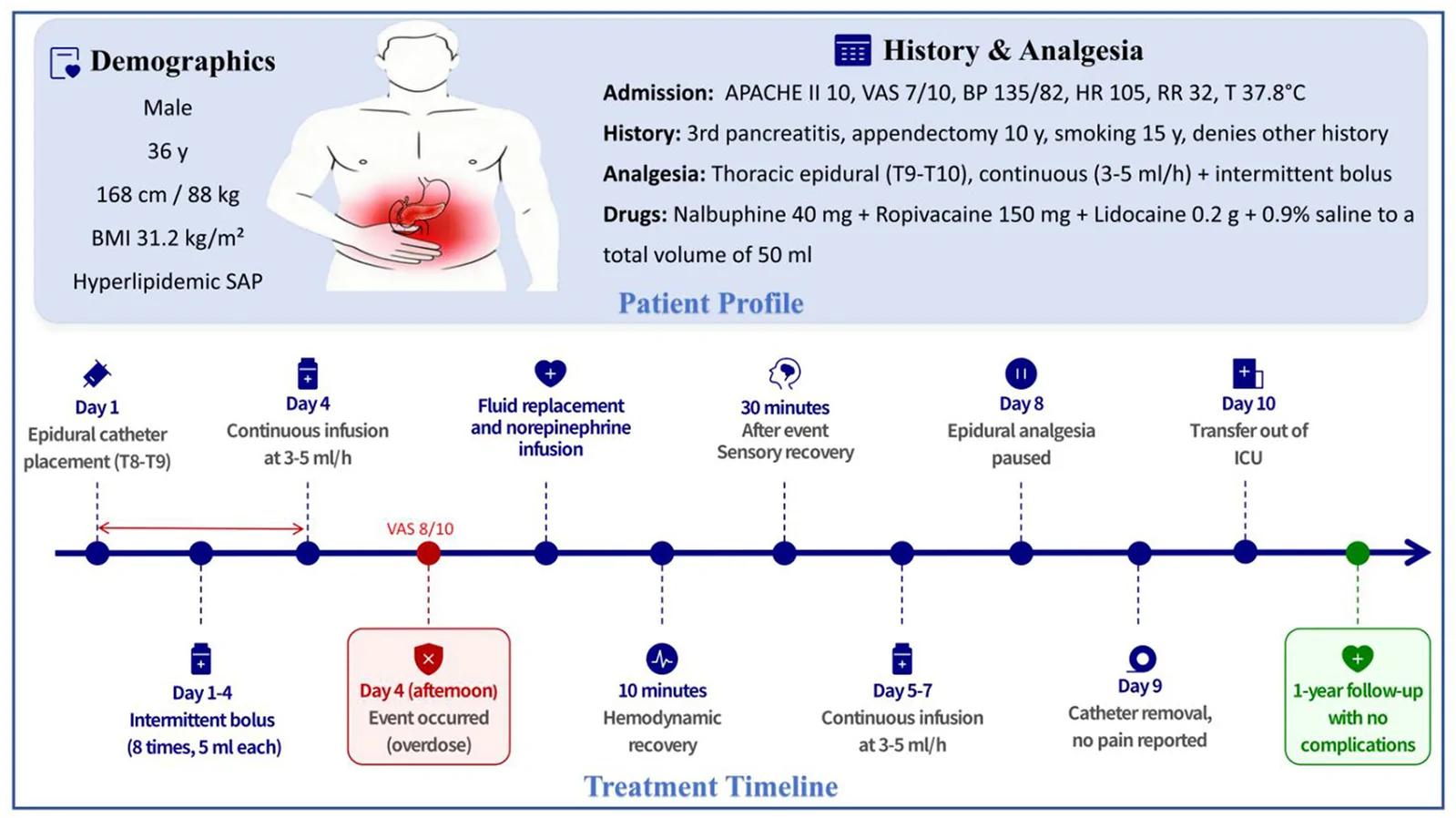
Patient profile and treatment timeline.

Daily neurological assessments after the event showed full recovery of lower limb muscle strength (grade 5) and normal sensory function, with no residual neurological deficits. The patient continued thoracic epidural analgesia without further complications. On day 8, epidural analgesia was paused, and the patient reported no significant pain. The epidural catheter was removed on day 9. On day 10, the patient was transferred from the ICU to the gastroenterology ward for continued pancreatitis management and was discharged without neurological sequelae. Long-term follow-up at 1 year revealed no related complications. The patient profile and treatment timeline are illustrated in Figure 2.

## 5 Discussion

### 5.1 Literature review and characteristics of this case

Medication errors in anesthesia and pain management are relatively uncommon; however, when they occur, the consequences can be severe. A narrative review covering the period from 1999 to 2019 reported 130 cases of neuraxial and peripheral nerve misconnections. The most frequent type was inadvertent injection of intravenous drugs into the epidural space, accounting for 29.2% (38/130), followed by epidural drugs mistakenly administered intravenously at 27.7% (36/130), and unintended intrathecal administration of intravenous drugs due to misconnection at 25.4% (33/130) (10). Such errors may result in serious complications, including spinal hematoma, infection, and cauda equina syndrome, and in severe cases can lead to spinal cord injury, paraplegia, seizures, or even death (11, 12).

Previous reports have documented a wide range of drugs inadvertently administered into the epidural space, including antibiotics, chemotherapeutic agents, potassium chloride, tranexamic acid (13), insulin (14), and oxytocin (15). Conversely, epidural medications such as local anesthetics and opioids may also be mistakenly administered via the intravenous route, indicating that route misidentification is a central mechanism underlying these errors (16). To mitigate this risk, the International Organization for Standardization introduced the ISO 80369 NRFit connector, which ensures physical incompatibility between neuraxial and intravenous systems (17). Additional preventive strategies, such as color coding and warning labels, have also been implemented to enhance medication safety. Nevertheless, these errors remain difficult to eliminate entirely. Borden et al. reported a case in which a tenfold overdose of epinephrine, caused by an error in preparing an epidural test dose, led to severe cardiac arrhythmia (18). This finding highlights that medication errors in neuraxial administration can result in significant hemodynamic instability and require heightened clinical vigilance.

The present case highlights an underrecognized mechanism, namely misinterpretation of pump alarm signals by nursing staff. This issue is closely related to alarm fatigue in ICU settings, which has been shown to increase nurse workload and endanger patient safety (19). An intravenous infusion pump was used to deliver analgesics via an epidural catheter, and repeated occlusion alarms were misinterpreted as failure of drug delivery. This led to continued bolus administration and resulted in local anesthetic overdose, hypotension, and bilateral lower limb numbness. This event was associated with insufficient understanding of device characteristics, limited clinical experience, and inadequate training. It underscores the risk of misinterpreting occlusion alarms in epidural administration and has important implications for improving the safety of nurse-led analgesia management. Adjusting occlusion alarm thresholds for epidural use may reduce unnecessary alarms, but carries risks.

### 5.2 Root cause analysis and event mechanism

**Figure 3:**
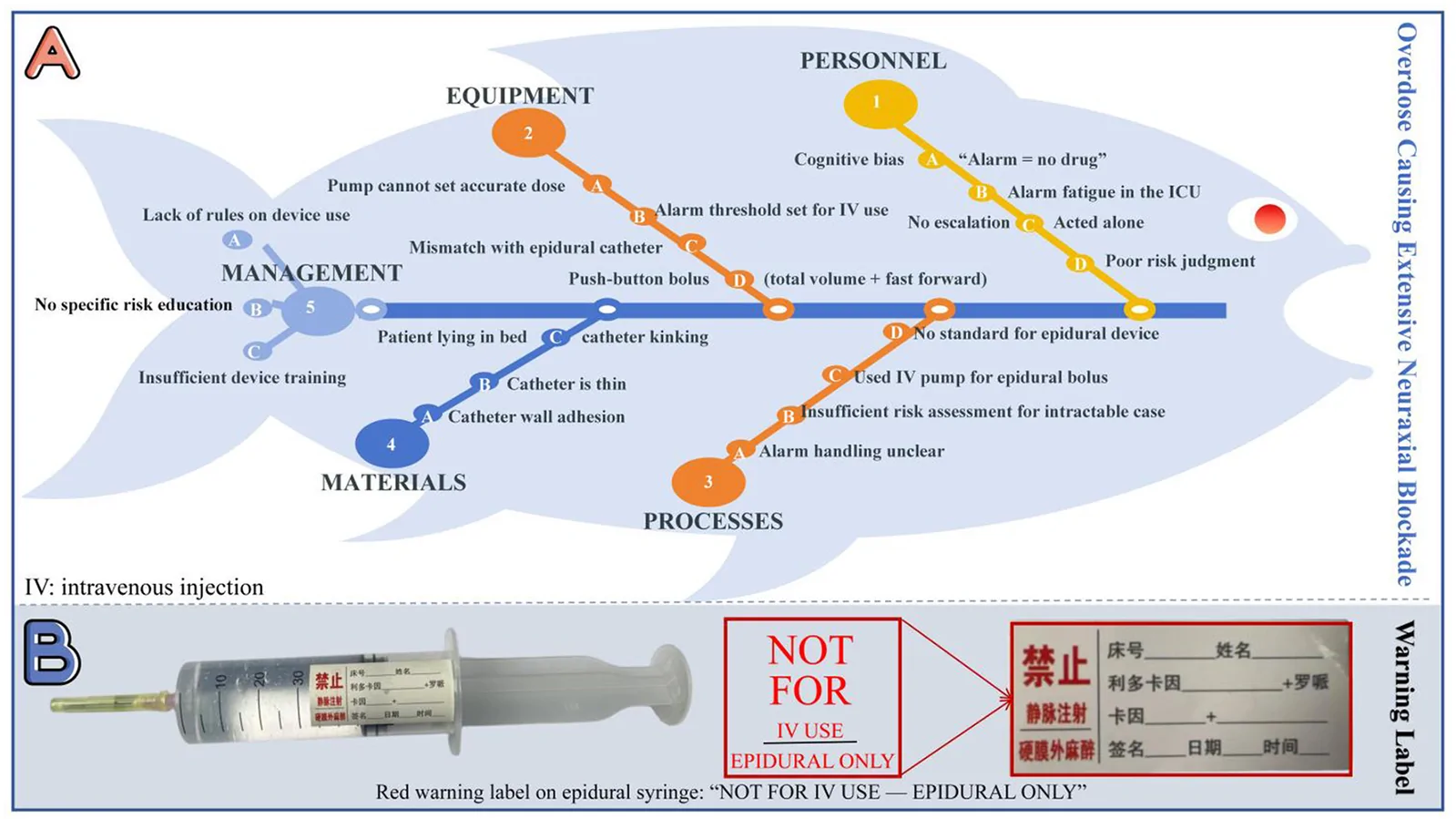
Fishbone diagram of root cause analysis and warning label. **(A)** Fishbone diagram; **(B)** Warning label on syringe.

As shown in the fishbone diagram (Figure 3A), an internal investigation using this method analyzed system defects across personnel, equipment, processes, materials, and management (20, 21). Under the nurse-led pain management model, nurses exhibited cognitive bias in interpreting “occlusion” alarms, mistaking them for “drug not delivered,” and handled alarms without consulting supervisors, which was an error, reflecting insufficient risk recognition and decision-making, and this may also be partly explained by alarm fatigue in the high-stress ICU environment, where frequent false alarms cause desensitization and delayed responses (22). Equipment factors included an outdated intravenous infusion pump unable to precisely set doses and relying on manual acceleration for bolus delivery; the pump was physically incompatible with the epidural catheter, and its alarm threshold, set for intravenous lines, could not distinguish high catheter resistance from complete occlusion. Process deficiencies included the absence of dedicated guidelines for epidural bolus administration, unclear alarm handling procedures, and lack of limits on bolus speed. Material factors involved the small-diameter epidural catheter, which, after several days of indwelling and combined with patient immobility, was prone to kinking or wall adhesion, increasing resistance. Management factors included the absence of institutional rules on device applicability and insufficient specialized training on equipment characteristics and risks.

The “occlusion” alarms were triggered by interactions between catheter properties and external factors: the small, soft epidural catheter was susceptible to kinking, twisting, or tip adhesion, and patient immobility further increased mechanical obstruction risk; additionally, blood clots, drug precipitation, and lipid deposits could narrow the lumen. In this case, the physiological high resistance of the epidural catheter was interpreted by the pump as an occlusion, while drug had partially entered the epidural space. Nurses, with limited experience, misjudged the alarm as “drug not entering or slow flow through narrow catheter” and continued repeated bolus administration. When the obstruction temporarily resolved, sudden pump pressure release caused hidden overadministration, with cumulative doses far exceeding safe epidural limits, ultimately resulting in extensive sympathetic blockade.

Clinically, hypotension, reflex tachycardia, and bilateral lower limb sensory deficits appeared 2 to 5 min after bolus, with the sensory block corresponding to T8–T9, indicating predominantly caudal spread without cranial extension to the C3–C5 phrenic nerve level, thereby avoiding respiratory muscle paralysis. Rapid fluid resuscitation and norepinephrine support restored hemodynamics and neurological function within 30 min without sequelae.

### 5.3 Lessons learned and system improvement

Medication errors have diverse root causes and require multifaceted prevention strategies. From this case, key lessons were identified: cognitively restructuring SAP pain management nurses by challenging the ingrained mindset that “an alarm implies failed drug delivery” and promoting correct understanding of the high-resistance characteristics of epidural catheters; implementing a four-step workflow of “alarm–assess–verify–execute,” pausing infusion during high-resistance alarms, investigating causes, and administering medication only after obstruction clearance; integrating device features and alarm mechanisms into core competency training with simulation exercises to enhance complex decision-making; and fostering a safety culture through system-level analysis, supportive second victim programs, non-punitive reporting, and clear visual warnings, such as red labels on syringes indicating “For Epidural Use Only—No Intravenous Administration,” which have proven effective (Figure 3B). Following this event, further improvements included replacing epidural pumps with controllable micro-infusion pumps, strengthening senior nurse supervision of junior staff, requiring that new nurses observe a minimum number of TEA pump administrations (e.g., 10 cases) performed by experienced nurses and receive approval before independent operation, prioritizing manual push, using a separate 5 mL syringe for hand-delivered bolus, or dedicated pumps, which are controllable micro-infusion pumps with features such as programmable dosing, mode-specific pressure thresholds, intervals greater than 2 h, and electronic logging capabilities (23, 24), enhancing pre-job assessments for epidural analgesia management, and refining reward criteria for voluntarily reporting near-miss events. Medication safety is not merely a technical issue but a systemic and cultural one; only such a multidimensional strategy can ensure safe nurse-led epidural analgesia management (25, 26). Post-event analysis indicated that the nurse acted in good faith to relieve the patient's pain. The critical issue was a system with multiple vulnerabilities rather than a single individual error. Regulatory measures, such as the U.S. Food and Drug Administration (FDA)'s review of confusing drug names, improved packaging, barcode verification, and patient education, have reduced errors, while in China, the “Three Checks and Seven Rights” framework emphasizes the correct drug, patient, dose, route, and timing. These principles have been instrumental in reducing medication administration errors in Chinese clinical settings.

## 6 Contributions and limitations

The value of this report lies in its comprehensive presentation of a nurse-led TEA management approach for critically ill SAP patients, demonstrating the combined use of continuous and intermittent infusion in individualized pain management. It also delineates the clinical chain of “alarm misinterpretation—pressure release—covert overdose—caudal-first spread” during intravenous pump epidural bolus, highlighting the risks associated with alarm misjudgment. Early recognition and intervention effectively reversed extensive sympathetic blockade caused by local anesthetic overdose, with the sensory block spanning T9–T12, caudal extension to L1–S5, limited cranial spread not exceeding T6, and sparing of the diaphragm area innervated by C3–C5, thereby preventing respiratory muscle paralysis. The root causes were identified as insufficient device knowledge, limited clinical experience, and lack of targeted training among nurses, providing a reference for early warning of similar events and optimization of nurse-led analgesia protocols. Limitations include its single-center case design, which precludes assessment of incidence or the influence of confounding factors such as drug concentration, infusion rate, and patient positioning, thus limiting generalizability.

### 6.1 Patient perspective

In this specific case, after the event, the medical team promptly informed the patient about what had happened and the measures taken. The patient expressed understanding and stated, “At that time, I just felt my legs suddenly become weak, and then I felt a bit dizzy. But the doctors handled it quickly, and I felt better soon after.” The patient was satisfied with the timely management by the medical team and actively cooperated with subsequent treatment. During follow-up, the patient reported complete recovery and expressed satisfaction with the treatment outcome.

## Data Availability

The raw data supporting the conclusions of this article will be made available by the authors, without undue reservation.
